# A Case of Injury by Lightning

**Published:** 1852-11

**Authors:** J. W. Spaulding

**Affiliations:** Victoria, Knox Co., Ill.


THE NORTH-WESTERN

MEDICAL AND SURGICAL JOURNAL.

NEW SERIES.

VOL. I. NOVEMBER, 1852. No. 7.

### ORIGINAL COMMUNICATIONS.

ARTICLE I.—*A Case of Injury by Lightning* by J. W. SPAULDING, M. D. Victoria, Knox Co., Ill.

IT seems to be the settled opinion of scientific men, that in injury by lightning, no burn is the direct consequence of the electric fluid, but that serious injuries are produced, such as contusion, laceration, or fracture. In many cases no external marks of violence exist. "I have not met with the account of any case where the appearance of a burn has been produced by the direct action of a stroke of lightning: for in those instances in which burns have been found upon the body, it appears that ignition of the clothes or articles of furniture had taken place, to which, alone, the burning was to be attributed."—(Taylor's Medical Jurisprudence, page 607.)

As an interesting case occurred in my practice (several years ago, which has been reported to the Stark Co. Medical Society, Ill.,) which strongly militates against the above views, I send it, and if you think it worthy of a place in your Journal, it is at your service.

On the 24th of June, 1843, there was a small cloud arose in the west, late in the afternoon, and it rained about fifteen minutes. During the shower there was one solitary clap of thunder, and one flash of lightning, which were nearly simultaneous. The lightning struck a cabin three miles from my residence; there were four persons in the cabin at the time, Mr. W., who stepped in to avoid the shower, and the woman of the house, and her two children.—Mr. W. was sitting on the left of the fire place, the woman with an infant in her lap, on the right, and a small boy on the bed, he being the only one that escaped injury.

The electric fluid passed down the chimney until it arrived at the log that formed the mantletree, where it perforated a piece of chinking, making a mortice about three inches long: another portion forced for itself a passage in the top of the log, making a groove of two inches in diameter, the main current perforated the floor in front of the hearth and disappeared in the ground.

The chinking on the opposite end from the chimney were all forced out, and boards thrown from the roof, the furniture and crockery thrown down and demolished.

Mr. W. and the woman were both struck down, and the woman remained in a state of unconsciousness for an hour, when she aroused and discovered Mr. W. lying on his face, almost entirely divested of his clothing. She turned him over, supposing him to be dead, and gave the alarm.

His wife and brother were soon on the ground, and found him lying in the position left by the woman. His buckskin pantaloons were torn into shreds, and forced into the crevices between the logs at the further end of the cabin; the rest of his clothing was torn from him, and the shirt, or a portion of it, ignited, producing a superficial burn on the shoulders.

The lightning struck him on the back part of the left parietal bone, ran down over the cervical and upper dorsal vertebræ, destroying the cutis between the scapula the size of a person's hand; it then passed to the left side, where the parts were extensively burned; it then passed over the ossa pubis, penis, scrotum, denuding them of the cuticle, and continued its course, in a serpentine direction, over the ham, down the outside of the leg, and perforated the boot near the heel, where it made its escape.

The whole track from his head to his heels left a burn about 3 inches wide, except where the destruction was much more severe.

There was a severe burn on the palmer side of the left arm, extending from the second phalanx of the thumb, to near the elbow. The right elbow was likewise severely burned over the olecranon.

The severer burns were baked to a crisp, and there was nothing ignited around him that could have produced so serious an effect, for the small portion of the shirt that burned left the marks about the shoulders, and was easily distinguished from the other injuries. Nothing else about the premises was scorched, leaving it self-evident that lightning and nothing else was the cause of those severe burns.

His brother, after dashing a pail of cold water on him, and placing him on a bed, came for me. Before my arrival, animation returned, and from his furious conduct his wife was fearful that he would seriously injure himself by jamming his head against the walls of the cabin, and she let him out upon the prairie.

I found him eighty rods distant, on his return, led by his wife, perfectly unconscious of all surrounding objects, and in a state of perfect nudity.

I relieved the woman, (who fainted from over excitement,) and led him to his house, where he was prevented from injuring himself until assistance arrived, when I bled him liberally, and he quieted down.

He remained quiet through the night, and sensibility slowly returned. In the morning he answered questions rationally, but had no knowledge of the cause of his present condition.

During the day, his pulse became sharp and frequent, his skin dry and hot, and his tongue coated. He was bled and purged, and the bleeding repeated daily for six or seven days, when the excitement was controlled.

The burns commenced suppurating in a few days, when they were treated with linseed and charcoal poultices, and the dead parts removed, in order to lessen the excessive fœtor.

The whole muscular portion of the obliquus externus was removed, without pain, exposing the fibres of the transversalis; the lower portion of the pectoralis major, was removed or sloughed, so as to expose a portion of the serratus magnus; the anterior edge of the latissimus dorsi was destroyed, and the origin of the glutei muscles, so as to expose the whole crest, and a portion of the dorsum of the ilium; and a portion of the crest exfoliated.

The whole of the soft parts of the anterior portion of the fore-arm, muscles, tendons, and radial artery, sloughed; and a portion of the radius four inches long, exfoliated.

There was an extensive slough of the right elbow, and numerous smaller ones in different localities.

During the suppurating stage he was kept on a rich, nourishing diet, and stimulants. The suppurative surfaces were dressed with skin or mild cerates, as indicated. He was able to be up in eight weeks, and in a short time the parts filled up by granulation, and cicatrized, leaving him crippled in the left hand.

